# Introductory patient communication training for medical physics graduate students: Pilot experience

**DOI:** 10.1002/acm2.13449

**Published:** 2021-10-28

**Authors:** Laura Padilla, Whitney Burton Meleski, Caitlin Dominick, Caroline Athing, Cassidy L. Jones, Dana Burns, Lauretta A. Cathers, Emma C. Fields

**Affiliations:** ^1^ Virginia Commonwealth University, Department of Radiation Oncology 401 College St Richmond Virginia USA; ^2^ Virginia Commonwealth University, School of Nursing Richmond Virginia USA; ^3^ Virginia Commonwealth University, College of Health Professions Richmond Virginia USA

**Keywords:** communication training, direct patient care, education, medical physics 3.0, patient communication

## Abstract

Despite medical physics becoming a more patient‐facing part of the radiation oncology team, medical physics graduate students have no training in patient communication. An introductory patient communication training for medical physics graduate students is presented here. This training exposes participants to foundational concepts and effective communication skills through a lecture and it allows them to apply these concepts through realistic simulated patient interactions. The training was conducted virtually, and eight students participated. The impact of the training was evaluated based on changes in both confidence and competence of the participants’ patient communication skills. Participants were asked to fill out a survey to assess their confidence on communicating with patients before and after the training. They also underwent a simulated patient interaction pre‐ and postlecture. Their performance during these was evaluated by both the simulated patient actors and the participants themselves using a rubric. Each data set was paired and analyzed for significance using a Wilcoxon rank‐sum test with an alpha of 0.05. Participants reported significantly higher confidence in their feeling of preparedness to interact with patients (mean = 2.38 vs. 3.88, *p* = 0.008), comfort interacting independently (mean = 2.00 vs. 4.00, *p* = 0.002), comfort showing patients they are actively listening (mean = 3.50 vs. 4.50, *p* = 0.005), and confidence handling challenging patient interactions (mean = 1.88 vs. 3.38, *p* = 0.01), after the training. Their encounter scores, as evaluated by the simulated patient actors, significantly increased (mean = 77% vs. 91%, *p* = 0.022). Self‐evaluation scores increased, but not significantly (mean = 62% vs. 68%, *p* = 0.184). The difference between the simulated patient and self‐evaluation scores for the postinstruction encounter was statistically significant (*p* = 0.0014). This patient communication training for medical physics graduate students is effective at increasing both the confidence and the competence of the participants in the subject. We propose that similar trainings be incorporated into medical physics graduate training programs prior to students entering into residency.

## 1 INTRODUCTION

Momentum is starting to build within the medical physics profession to transform itself from being mostly behind‐the‐scenes to emerging as a more visible and patient‐facing member of the healthcare team within its technical role.^1^, ^2^, ^3^, ^4^, ^5^ As the landscape of medical physics evolves and the profession becomes more involved in direct patient care, the training provided for students entering the field needs to be revised to prepare them for their potential impending duties, such as one‐on‐one meetings with patients during physics consults.^1^ In addition to technical expertise, successful interactions with patients will require physicists to be able to communicate in a clear, engaging, and empathetic manner. Although communication skills are sometimes viewed as innate and unteachable, it has been well documented in the literature that they can be effectively taught and developed.^6^, ^7^, ^8^, ^9^, ^10^, ^11^, ^12^ Thus, it is unsurprising that communication skills have been embedded into the medical school curriculum to allow future doctors ample time to work on and mature their skill set prior to independent practice.^13^, ^14^, ^15^ Current medical physics training in patient communication is limited, although programs have been successfully implemented for residents and practicing medical physicists.^16^, ^17^ However, there is currently no training being provided at the graduate program level. Exposing our learners to such curriculum early in their career would give them the opportunity to acquire, repeatedly practice, and improve their communication skills under controlled and safe conditions prior to independent practice. This deliberate integration and practice is essential for effective and long‐lasting learning.^18^, ^19^ This article presents the first patient communication training curriculum, to the authors’ knowledge, specifically designed for medical physics graduate students.

## 2 METHODS

This study was reviewed by the Institutional Review Board at Virginia Commonwealth University and deemed exempt. The training was offered to graduate students of all levels and it was independent of any course, and thus, had no impact on the participants’ grades or standing in the program.

### 2.1 Curriculum design

A curriculum was created to introduce medical physics graduate students to effective techniques for patient communication. It was developed by a medical physicist in conjunction with a radiation oncologist, social workers, and two experts in simulated patient interactions. This training focused on patient communication in radiation oncology. The curriculum was designed following Kolb's experiential learning theory where a learner is presented with a new experience, given time to reflect on the experience, conceptualize what they have learned from it and how it can help them in future situations, and given the chance to apply those new findings to a new situation.^20^ Our program consisted of four parts: preparation (patient testimonial, introductory presentation, first simulated patient encounter), instruction (didactic lecture), application (second simulated patient encounter), and final reflection. These are explained in more detail below.

#### 2.1.1 Preparation

Prior to any activities, participants were asked to fill out a pre‐intervention survey to assess their confidence level with patient communication. The questions on this survey were created to assess their comfort level with interacting with patients overall, as well as to check their level of confidence utilizing specific effective communication strategies such as active listening, showing empathy, and using appropriate language for a given patient. Questions about these specific strategies were included because they were part of the material covered during the didactic lecture.

Due to the lack of clinical experience of most graduate students, as expected from the stage in their training, it was important to begin by exposing them to what patients experience during the radiotherapy treatment process. Participants were asked to watch four videos corresponding to some relevant time‐points in radiotherapy (initial consult, simulation, start of treatment, end of treatment). These videos are available on YouTube and were recorded by a young breast cancer patient documenting her journey.^21^ These videos were selected because they were not sponsored or created by a particular institution, and they gave an unscripted first‐hand description, through this patient's personal video blog, of what a patient goes through at each stage of their cancer treatment with open and candid narration. Participants were then given prompts to reflect on the videos and asked to write down their thoughts. The prompts were designed to help them investigate how what they saw challenged their perception of the patient experience in radiation oncology and how they thought they could personally, as medical physicists, have an impact on patient experience in the future.

To ensure all participants had the radiation oncology knowledge necessary for the subsequent simulated patient encounters, we designed an introductory presentation to review this information. This presentation briefly covered the typical steps a patient goes through when undergoing radiotherapy. This was important to ensure participants were not distracted by fears of having inadequate technical knowledge. In addition to providing them with this basic information, we emphasized that the focus of simulated patient interactions would be on their communication skills and that their technical knowledge would not be evaluated at all. The presentation also provided participants with an overview of what simulated patient encounters are and their purpose. The last step in the preparation portion of the training was for the participant to experience their first simulated patient encounter. Simulated patients are actors trained to play the role of a patient in a given scenario. These actors receive a description of their role and the scenario they will be portraying. The medical physicist and radiation oncologist in our group, along with staff from the simulation center in our institution, met with the patient actors to train them in their role. This first patient encounter consisted of an affable prostate patient meeting with their medical physicist prior to their CT simulation appointment to get an overview of what CT simulation is, why it is needed, how it fits into the radiotherapy process, and answer any questions that arise. Encounters were 10 min long. The simulated patients were asked to evaluate the participant based on a rubric provided by the authors designed to assess behaviors related to effective communication skills. This rubric was created with the guidance of the two experts on simulated patient encounters among the authors and it was based on the communication rubric developed through the Macy Initiative in Health Communications.^22^ This rubric was reviewed with the patient actors during training to clarify any questions about the content. No further interrater reliability training was performed, as the potential subjectivity of the simulated patients’ impressions was taken to be a good surrogate for patient variability during real clinical interactions. Simulated patients were also asked to give verbal feedback to each individual participant following their encounter. Participants were asked to self‐evaluate using the same rubric after the encounter.

#### 2.1.2 Instruction

This portion consisted of a 1.5‐h lecture developed and led by the social workers. Its content encompassed the foundational knowledge and strategies needed to build effective communication skills, and a communication framework to be used as a tool to structure encounters when interacting with patients. The selected framework used for this training^23^ is consistent with that utilized in the current patient communication training program that exists for residents and practicing medical physicists.^16^, ^17^

#### 2.1.3 Application

After being presented with the basics of effective patient communication, learners were then asked to participate in a second patient encounter to apply the knowledge they had gained from the lecture. The premise for the application encounter was the same as for the preparation encounter, a meeting between the patient and the medical physicist prior to CT simulation. However, the simulated patient in this encounter was a breast cancer patient instructed to be more inquisitive during the interaction. Simulated patients were asked to evaluate the performance of the participants using the same rubric as in the preparation encounter, and to give individual participants verbal feedback after each interaction. Participants were also asked to self‐evaluate after this experience. Once all participants finished their encounter and self‐evaluation, the medical physicist and radiation oncologist met with the group to debrief about their experience and share common lessons.

#### 2.1.4 Final reflection

To conclude, the training participants were asked to write a reflective piece on their experience during this training following prompts provided by the authors. The aim of the final reflection was to help them identify areas of comfort and improvement as well as to think about how the skills learned during the training could help them in their career as medical physicists. Participants were also asked to complete the survey to assess their confidence in patient communication again.

### 2.2 Data analysis

The effectiveness of this curriculum was evaluated both for its impact on self‐reported confidence by the learners and on their competence in patient communication as assessed by both the simulated patients and self‐evaluation by the participants. Surveys designed to assess confidence consisted of questions to be answered based on a Likert scale from 1 (strongly disagree) to 5 (strongly agree). Simulated patient encounter rubric answers were also based on Likert‐scale answers from 0 (not at all) to 3 (completely). Survey responses collected during the preparation and final reflection portions were paired for analysis to assess the impact of training on patient communication confidence. Changes were evaluated for significance on a per‐question basis using a one‐tailed Wilcoxon rank‐sum test with an alpha of 0.05. Rubric answers for simulated patient encounter evaluations for each participant were combined and converted into a single percentage value for each encounter. Preparation and application encounter results were paired for analysis to assess the impact of the training on communication competence. Pre‐ and postinstruction changes were evaluated for significance using a one‐tailed Wilcoxon rank‐sum test with an alpha of 0.05 for the simulated patient, and self‐evaluation scores, separately. Self‐evaluation and simulated patient scores for the preparation and application encounters were also compared between the two groups (simulated patient vs. self‐evaluation scores for each encounter). Since it was unclear whether a group would assign scores higher than the other, the evaluation for significance was performed using a two‐tailed Wilcoxon rank‐sum test with an alpha of 0.05.

## 3 RESULTS

The training was given between March and May of 2020 and had 10 participants (one certificate, three Masters, three PhD, one postdoctoral student, and two residents). Since the curriculum was designed specifically for learners who have not started their clinical training yet, resident data were not included in the analysis (final *n* = 8). Due to the coronavirus pandemic, all portions of the training (except the introductory presentation completed in early March) were given virtually.

**FIGURE 1:**
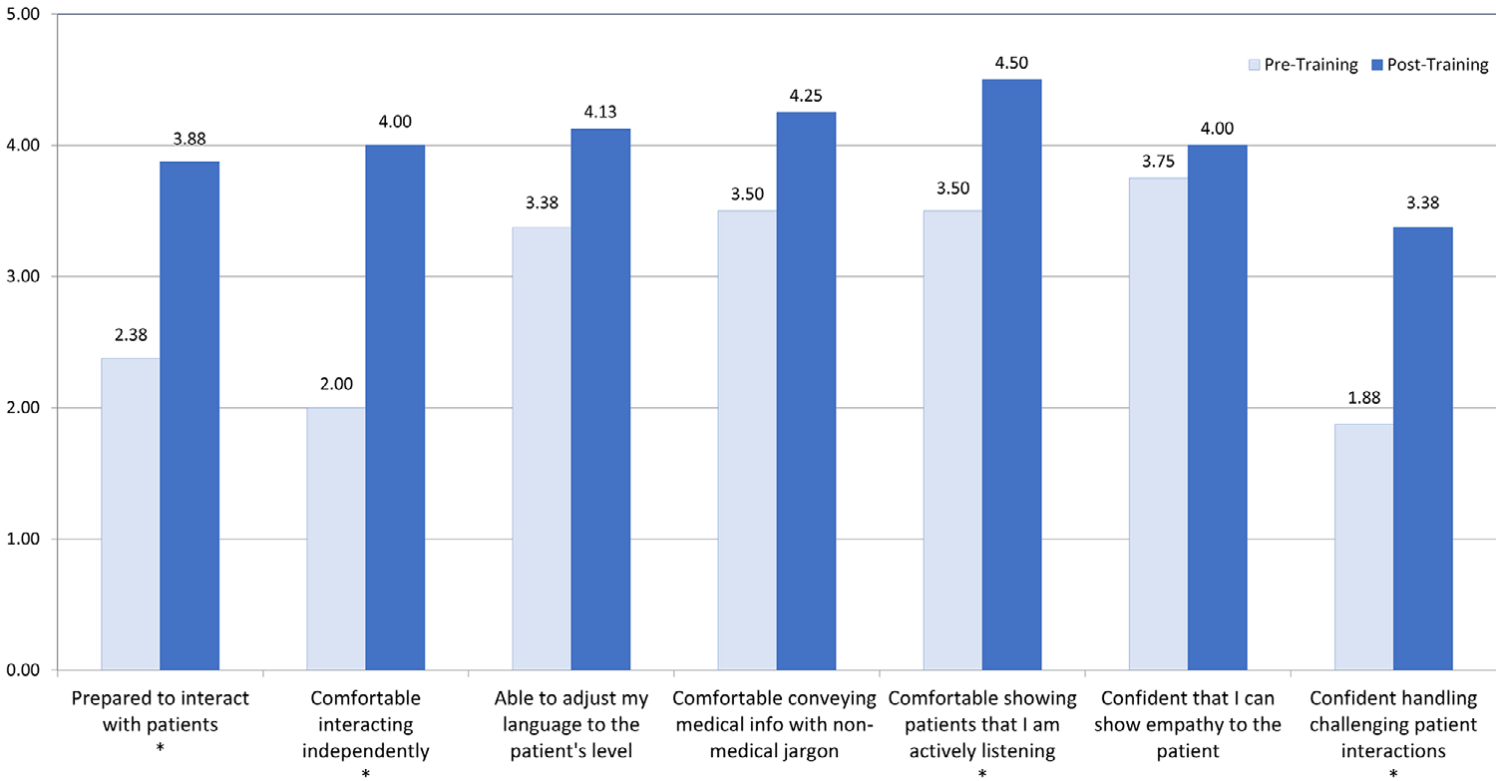
Pre‐ and posttraining survey results to assess participants' confidence in patient communication. Statistically significant changes (*p* < 0.05) are denoted with an asterisk at the bottom of the column label. Mean values shown above each column

Self‐reported confidence on patient communication skills increased after participating in the training (see Figure 1). This was statistically significant for their feeling of preparedness to interact with patients, comfort interacting independently, comfort showing patients they are actively listening, and confidence handling challenging patient interactions. Increases were seen across all aspects investigated but the rest were not statistically significant.

**FIGURE 2:**
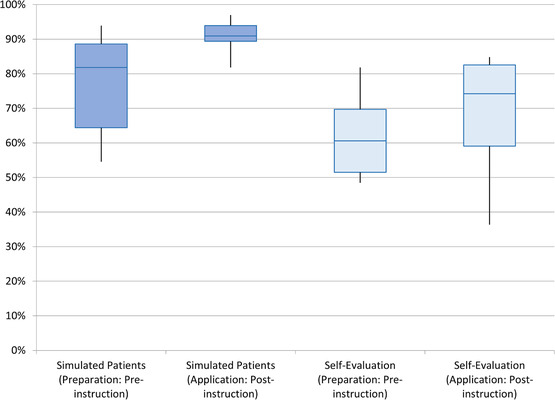
Boxplots of the simulated patient encounter scores distribution for the pre‐ and postinstruction interactions

Performance scores assigned by the simulated patients showed a statistically significant increase (mean_pre _= 77%, mean_post _= 91%, *p* = 0.022) between the preparation and application encounters (pre‐ and postinstruction). In contrast, self‐evaluation scores for the participants increased between the two encounters, but the change was not statistically significant (mean_pre _= 62%, mean_post _= 68%, *p* = 0.184). Simulated patients scored the participants’ performance significantly higher in the application encounter compared to the participants’ self‐evaluation scores (*p* = 0.0014). Figure 2 illustrates the distributions of the scores by the two cohorts.

## 4 DISCUSSION AND CONCLUSIONS

The work described here presents the first patient communication training designed specifically for medical physics graduate students. This training provides students, regardless of their graduate year or intended terminal degree (Masters, PhD, or certificate), with an initial introduction to effective patient communication techniques and a first experience interacting with a patient as a physicist in a realistic, controlled, and safe environment. This training is short and should by no means be the only exposure students get to this topic during their education, as continuous repetition and practice are important to develop robust communication skills.^19^, ^24^ Although the specifics of its integration into existing medical physics programs is highly dependent on a given program's structure, culture, and focus, it would be valuable to expose students to this training as early in their graduate studies as possible. This would give students ample opportunity to participate in follow‐up programs, as they get developed, to help them maintain and expand their communication skills. Future patient communication training programs could leverage the foundation established by introductory trainings to build on more advanced topics such as shared decision making, difficult conversations, communication with pediatric patients and their families, unconscious bias and how it affects interpersonal communication, etc.

Despite the compressed design of the training, it contains high quality content and activities based on evidence‐based strategies. We recruited social workers to drive the didactic content of the training as the pillar of their profession is effective interpersonal communication skills, and they have invaluable experience interacting with patients. We designed the training to be heavily experiential due to its proven value for teaching these skills.^12^, ^18^, ^19^, ^20^ We also included self‐reflection activities as these complement experiential learning and are a helpful tool for communication training.^25^, ^26^, ^27^, ^28^ Although the focus of this training was radiation oncology, the effective patient communication skills taught through it are universal and would be applicable to any medical physics specialty. However, it is necessary to develop examples and patient encounters that represent diagnostic and nuclear medicine scenarios. Effective patient communication skills are beneficial to all specialties in medical physics and thus should be represented accordingly, especially at the graduate level. Since patient communication training is a new practice in the field, the authors purposefully selected the same communication framework^23^ for this training as what is being used by the University of California, San Diego to train resident and practicing medical physicists.^16^, ^17^ This was done to promote consistency in the field as we explore how to best incorporate this topic within the medical physics curriculum.

As evidenced by the results, this short program served to improve both the confidence and competence of the participants. Although participants reported having significantly higher confidence in preparedness to interact with patients and comfort interacting independently after the training, their self‐evaluation scores in the postinstruction patient encounter were not significantly higher than in their pre‐instruction encounter. Interestingly, simulated patient actors scored the participants’ performance higher in both encounters, but significantly so in the postinstruction one. This may indicate that participants potentially evaluate their performance more harshly or cannot accurately read what the patient experiences during the interaction. Additionally, the second encounter might have been perceived to be more difficult by the participants since the simulated patient was instructed to be more inquisitive than in the first scenario. This may have affected self‐evaluation scores. Although designed differences in patient gender, age, and disposition between the first and second encounters introduce confounding factors into the comparison of pre‐ and postinstruction competence results, it was important to expose participants to a different situation for each case, and for the second one to be more challenging than the first. This served two purposes: first, we were able to more confidently determine that their change in performance was due to application of newly learned communication strategies rather than increased familiarity with the same case, and second, it provided a richer experience for the participants by allowing them to practice communicating with different types of patients. To better characterize the full extent of this curriculum's impact, it will have to be implemented at more institutions to obtain a larger sample size and more diverse cohort.

There are some limitations in implementing this workshop on a national level. Simulated patient encounters require knowledge of how to design the activity, train the actors, and obtain funding to pay for them. Fulfilling the funding requirement may be difficult given current departmental budgets. Although these encounters provide learners with a realistic experience for training, alternative formats such as role play between participants or other faculty, may be more viable and should be investigated as they are still valuable experiential learning tools.^29^ Additionally, if a program does not have social workers who are able to aid in the implementation of the training, the authors encourage the readers to try to identify other members of the institution with medical interpersonal communication expertise or to contact the authors to obtain the didactic materials.

Finally, we originally planned to give this training completely in‐person. As the roll out was interrupted by the COVID‐19 pandemic, the simulated patient encounters and lecture had to be given virtually. Although the basics of effective patient communication are applicable to both the in‐person and online settings, certain aspects (such as eye contact—should one look at the screen to see the patient or at the camera to better simulate eye contact?) may be different in the two settings. This was not explicitly addressed in the lecture given in this training and may be a good addition for future versions. On the other hand, the unexpected virtual format of this training had its benefits. Although participants did not get to experience an in‐person interaction, they got to practice communication in the telemedicine setting, which may be more common in the future postpandemic.^30^, ^31^, ^32^ Also, since the training proved to be effective even when given online, this allows us to consider creating a virtual training network for patient communication education in medical physics that is not institution dependent. This would alleviate some of the current barriers in incorporating this training into institutions where the faculty body does not feel comfortable leading such training or does not have the bandwidth, funding, resources, or support to develop institution‐specific curricula for it. Although patient communication is not currently explicitly included as an item in the CAMPEP core graduate^33^ or residency curriculum lists for accreditation^34^ or on the AAPM guidelines for graduate^35^ or clinical medical physics residency programs,^36^ as our profession evolves it may become a necessary skill. Furthermore, professionalism and/or interpersonal and communication skills are explicitly listed in those reports, and although this training is geared specifically towards patient communication, the principles of effective communications presented in it are applicable to any interpersonal interaction.

## CONFLICT OF INTEREST

The authors have no conflicts of interest to report pertaining this work.

## AUTHOR CONTRIBUTIONS

All authors made significant contributions to this work, and the corresponding author has checked with each listed contributing author for permission to submit the manuscript in its current form to JACMP.
